# Older people's out-of-home mobility and wellbeing in Australia: Personal, built environment, and transportation factors associated with unmet mobility needs

**DOI:** 10.3389/fpubh.2023.1121476

**Published:** 2023-02-20

**Authors:** Tracey Ma, Conrad Kobel, Rebecca Ivers

**Affiliations:** ^1^School of Population Health, University of New South Wales, Sydney, NSW, Australia; ^2^Australian Health Services Research Institute, University of Wollongong, Wollongong, NSW, Australia

**Keywords:** healthy aging, transport mobility, community mobility, healthy city, age-friendly city

## Abstract

Out-of-home mobility is fundamental to older people's wellbeing and quality of life. Understanding the unmet mobility needs of older people is a necessary starting point for determining how they can be supported to be mobile. This study estimates the extent of unmet mobility needs among older Australians and identifies the characteristics of those most likely to report unmet mobility needs. Analysis was conducted on nationally representative data of 6,685 older Australians drawn from the 2018 Survey of Disability, Aging and Carers conducted by the Australian Bureau of Statistics. Twelve predictor variables from two conceptual frameworks on older people's mobility were included in the multiple logistic regression model. Twelve percent (*n* = 799) of participants had unmet mobility needs, and associated factors significant in multivariable models included being among the “young-old”, having a lower income, having lower levels of self-rated health, having a long-term condition, being limited in everyday physical activities, experiencing a higher level of distress, being unlicensed, having decreased public transport ability, and residing in major cities. Efforts to support older people's mobility must make equity an explicit consideration, reject a one-size-fits-all approach, and prioritize the accessibility of cities and communities.

## 1. Introduction

Older people's “ability to move about the city” for the purposes of “social and civic participation and access to community and health services” is paramount to, what the World Health Organization calls, an age-friendly society (1). Indeed, older people's out-of-home mobility is an important contributor to their wellbeing and quality of life (2–4), enabling their access to activities that facilitate health, such as health care services (5), and support their connection to community, such as leisure and social activities (6, 7). Moreover, regardless of the types of out-of-home activities pursued, the act of getting out and about is valued by older people and perceived to be a contributor to their wellbeing (8).

However, while out-of-home mobility is fundamental to the wellbeing of older people, older people's life circumstances pose challenges to their mobility. On average, older people have more leisure time, expanding their opportunities for out-of-home activity participation and out-of-home mobility (9, 10), but less financial resources, which can restrict those opportunities (9). Within this group though, there is much heterogeneity—more so than among young and middle-aged adults—due to individually-specific age-related changes in activity patterns, daily routines, and physiological and psychosocial processes (11, 12). As such, whole-of-population approaches to studying mobility, such as travel behavior modeling aimed at system-level planning, are insufficient for understanding older people's mobility (9). Rather, explicit attention is needed to understand the intricacies of mobility in older age and identify the unique considerations for supporting older people to be mobile.

Existing research on older people's mobility focuses heavily on travel behavior, providing a rich knowledge base for understanding the modes of transportation used (10, 13–15), the factors affecting their use (15–19), and the details of their use (14, 18, 20). Such information is useful for understanding how to support older people's existing modal choices and travel patterns and/or facilitate modal shifts. For example, information on average walking distances of older people may motivate changes to the urban environment to facilitate more active transport use by older people for trips within that distance, with physical activity benefits in mind (13). Likewise, understanding the barriers to public transport use among older people who chose to travel by car can support initiatives to make public transport more attractive to them, especially as they transition away from driving (15). The limitation of such information, however, is that it only captures the segment of the older population who is already mobile and theoretically reaping the wellbeing benefits associated with out-of-home mobility.

Another focus of research on older people's mobility is directed at understanding and identifying transport disadvantage, typically using indicative measures (21, 22) and often in select geographic locations (13, 14, 17, 21, 22). Transport disadvantage research complements travel behavior research by focusing on those not already mobile, at all or to the fullest extent, due to out-of-home mobility difficulties. Transport disadvantage can be indirectly inferred from trip rates (23), household spending on fuel (23), distance or travel time to services (21), levels of car ownership (24), and public transport service levels (25–28). However, while these measures are indicative, they do not directly measure if and the extent to which older people experience out-of-home mobility difficulty. Households that would otherwise be considered “transport disadvantaged” based on the indicators of income and car ownership are not necessarily disadvantaged, as household members may make deliberate decisions about their mobility and location and be well situated to manage their circumstances (24). Indicative measures of “transport disadvantage”, therefore, may not necessarily correspond with actual experiences of out-of-home mobility difficulty.

An alternative approach to examining older people' out-of-home mobility difficulty is to focus on unmet mobility needs. Unmet mobility needs are mobility needs that are unfulfilled due to difficulties accomplishing journeys or activities (29). When self-reported, it captures actual experiences of out-of-home mobility difficulty (30). Studies of older people in England, Norway, Finland, and the United States have examined unmet mobility needs using self-reported measures from nation-wide surveys (9, 30–33). Building on these international studies, this study aims to estimate the extent of unmet mobility needs in the older Australian population and identify the characteristics of older people who are most likely to have unmet mobility needs. Using self-reported measures from a nation-wide survey, this study regards unmet mobility needs to exist when people indicate that they would like to leave home more often than they actually do. This study extends earlier work by using two externally developed conceptual frameworks to guide variable selection and applying them to the largest sample size yet.

## 2. Materials and methods

Analysis was conducted on data from 6,685 community-dwelling older people (age 65 years or older) from the 2018 Survey of Disability, Aging and Carers (SDAC). The SDAC is a cross-sectional population survey conducted by the Australian Bureau of Statistics (ABS) (34). This survey was conducted from July 2018 to March 2019. The SDAC collects information from people with a disability, older people, and carers of older people and people with disabilities. Households are randomly selected and represent others in that area, in that State, and in Australia. Participation is mandatory for selected households to ensure a representative sample, as per the Census and Statistics Act 1905. Participation involves a face-to-face survey interview with an ABS interviewer. Participants were included in this analysis if they provided complete data. As this study uses secondary data, ethical approval of research was not required.

Analysis involved a multiple logistic regression model in which the outcome variable indicating unmet mobility needs was operationalized as “whether leaves home as often as would like”. Variable values were either “leaves home as often as would like” (i.e., No, there are no unmet mobility needs) or “leaves home, but not as often as would like” (i.e., Yes, there are unmet mobility needs). Twelve predictor variables were included in the model: age, sex, marital status (whether legally or de facto married), main language at home, weekly income, self-rated health, presence of a long-term condition, whether limited in everyday physical activities, level of distress (based on the Kessler Psychological Distress Scale), license status, public transport ability, and residential location.

The inclusion of these variables were supported by two conceptual frameworks: Luiu's framework for assessing unmet travel needs in later life (35) and Webber's framework of the determinants of mobility in older age (36) (see Table 1). Luiu's framework (35) was developed specifically to guide investigations into unmet mobility needs in later life with the suggestion that consideration of all five domains is necessary. These domains were derived from a three-step process: a literature review identifying 14 studies directly addressing factors affecting unmet mobility needs, a methodology assessment of each study, and a content analysis of each study for themes influencing mobility in later life. Webber's framework (36) was developed to overcome discipline-specific and disjointed portrayals of mobility in older age by proposing a comprehensive and interdisciplinary framework that links multiple categories of mobility determinants with multiple life-space locations with multiple means of mobility. It comprises five categories of determinants and has been found to acceptably fit data from a US national sample of older adults (37).

**Table 1:** Predictor variables.

	**Luiu's framework for assessing unmet travel needs in later life** **(****35****)**					
	**Demographics**		**Health and wellbeing**		**Built Environment**	**Transportation**
Age	X					
Sex	X					
Marital status	X					
Main language at home	X					
Weekly income		X				
Self-Rated health			X			
Long-Term condition			X			
Limited in everyday physical activities			X			
Level of distress				X		
License status						X
Public transport ability						X
Residential location					X	49pt
	**Gender, culture, and biography**	**Financial**	**Physical**	**Psychosocial**	**Environmental**	
	**Webber's framework of the determinants of mobility in older age** **(****36****)**					

The glm() function with a logit link function was used to fit the model using the RStudio (v1.1.423) interface for R (v4.0.2). Model results are presented using the tbl_regression() function of the {gtsummary} package (38), in which odds ratios (OR) were calculated by exponentiating model coefficients. The 95% confidence intervals (CI) calculated by the tbl_regression() function are consistent with those generated using the confint() function, which produces CIs based on the log-likelihood function. Results were considered statistically significant if the corresponding CIs do not include the null value. The Nagelkerke pseudo R-squared value was 0.3. The presence of multicollinearity was checked using the Variance Inflation Factor (VIF) with a cut-off of five. Multicollinearity was not present as the highest VIF value was 1.4.

## 3. Results

The majority of study participants were aged 65–75 (60%), married (64%), spoke English as their main language at home (93%), and had a weekly income of $500 or less (60%). There were slightly more females (52%) than males. Most of them had a long-term condition (87%), experienced low to moderate levels of distress (90%), and were not limited in everyday physical activities (80%). A similar proportion had excellent or very good self-rated health (49%) or good or fair self-rated health (47%). Most participants were licensed (89%) and were able to take all forms of public transport (88%). Participants primarily resided in major cities (62%). Of the 6,685 people included in the study, 96% (*n* = 6,417) left home in the last 2 weeks. Sample characteristics are presented in Table 2.

**Table 2:** Sample characteristics.

	***N =* 6,685**
**Age**	
65–74	4,000 (60%)
75–84	2,130 (32%)
85 and up	555 (8%)
**Sex**	
Male	3,223 (48%)
Female	3,462 (52%)
**Marital status**	
Married	4,246 (64%)
Not married	2,439 (36%)
**Main language at home**	
English	6,245 (93%)
Other	440 (7%)
**Weekly income**	
More than $500	2,650 (40%)
$500 or less	4,035 (60%)
**Self-rated health**	
Excellent or very good	3,248 (49%)
Good or fair	3,138 (47%)
Poor	299 (4%)
**Long-term condition**	
No	882 (13%)
Yes	5,803 (87%)
**Limited in everyday physical activities**	
No	5,374 (80%)
Yes	1,311 (20%)
**Level of distress**	
Low to moderate	6,001 (90%)
High to very high	684 (10%)
**License status**	
Licensed	5,973 (89%)
Not licensed	712 (11%)
**Public transport ability**	
All forms	5,873 (88%)
Some forms	262 (4%)
No form	550 (8%)
**Residential location**	
Major city	4,168 (62%)
Inner regional	1,677 (25%)
Other	840 (13%)
**Unmet mobility needs**	
No	5,886 (88%)
Yes	799 (12%)

Of the 6,685 people included in the study, 799 (12%) reported unmet mobility needs. Among those with unmet mobility needs, more were among the “young–old” (aged 65–74) (56%), female (56%), married (56%), English speaking (91%), and had a weekly income of $500 or less (74%). Additionally, those with good or fair self-rated health (62%), who had a long-term condition (96%), who were limited in everyday physical activities (52%), and who experienced low to moderate levels of distress (65%) were more represented among those with unmet mobility needs. There was also a greater proportion of people with unmet mobility needs who were licensed (74%), were able to take all forms of public transport (65%), and who live in major cities (65%).

Results of the regression analysis are presented in Table 3, with significant values in bold. The model indicates that, when adjusted for other predictors in the model, the following are significantly associated with having unmet mobility needs: being among the “young–old” (aged 65–74), having a lower weekly income, having lower levels of self-rated health, having a long-term condition, being limited in everyday physical activities, experiencing a higher level of distress, being unlicensed, having decreased public transport ability, and residing in major cities. Age, sex, marital status, main language at home, and weekly income are all independently associated with having unmet mobility needs, with the exception of the middle age bracket. However, only age and weekly income remain significant when adjusted. Moreover, when adjusted, their estimated effect diminishes and, for some, reverses direction. Self-rated health, presence of a long-term condition, whether limited in everyday physical activities, and level of distress remain significantly associated with having unmet mobility needs after adjustment, but with a greatly diminished effect. Likewise for license status and public transport ability. For residential location, the “inner regional” value becomes significant after adjustment.

**Table 3:** Regression analysis of unmet mobility needs.

	**Unadjusted**		**Adjusted**	
	**OR**	**95% CI**	**OR**	**95% CI**
**Age**				
65–74	–	–	–	–
75–84	1.14	0.97, 1.34	**0.82**	**0.68, 0.99**
85 and up	1.39	1.08, 1.79	**0.65**	**0.47, 0.89**
**Sex**				
Male	–	–	–	–
Female	1.19	1.02, 1.38	0.96	0.81, 1.14
**Marital status**				
Married	–	–	–	–
Not Married	1.45	1.25, 1.68	1.13	0.95, 1.35
**Main language at home**				
English	–	–	–	–
Other	1.41	1.07, 1.83	0.79	0.57, 1.08
**Weekly income**				
More than $500	–	–	–	–
$500 or Less	1.99	1.69, 2.35	**1.39**	**1.15, 1.68**
**Self-rated health**				
Excellent or very good	–	–	–	–
Good or Fair	3.84	3.18, 4.66	**2.13**	**1.73, 2.64**
Poor	22.84	17.29, 30.27	**4.30**	**3.06, 6.04**
**Long-term condition**				
No	—	—	—	—
Yes	4.05	2.87, 5.92	**1.55**	**1.08, 2.31**
**Limited in everyday physical activities**				
No	—	—	—	—
Yes	6.10	5.22, 7.13	**2.36**	**1.95, 2.86**
**Level of distress**				
Low to moderate	—	—	—	—
High to very high	7.38	6.18, 8.81	**3.15**	**2.56, 3.87**
**License status**				
Licensed	—	—	—	—
Not licensed	3.63	3.02, 4.35	**1.79**	**1.40, 2.26**
**Public transport ability**				
All forms	—	—	—	—
Some forms	5.09	3.86, 6.66	**1.95**	**1.41, 2.68**
No form	5.36	4.39, 6.52	**2.14**	**1.66, 2.75**
**Residential location**				
Major city	–	–	–	–
Inner regional	0.90	0.75, 1.07	**0.81**	**0.66, 0.98**
Other	0.77	0.60, 0.98	**0.61**	**0.46, 0.80**

Bold values are statistically significant.

## 4. Discussion

Using 2018 data from a population-wide survey of 6,685 older people, this study estimated the extent of unmet mobility needs in the older Australian population and identified the characteristics of those most likely to report unmet mobility needs. This study found that 12% of participants had unmet mobility needs¾ reporting that they would like to leave home more often than they actually do¾, despite the vast majority (96%) of participants already demonstrating some degree of mobility, having left home in the last 2 weeks.

Of the demographic variables, younger age and lower weekly income were found to be independently associated with unmet mobility needs. The presence of an age effect contrasts with similar studies in the United States (30), Norway (31) and England (32), which found no significant age effect after adjustment. The direction of the age effect might be explained by the “young–old” having more out-of-home mobility needs than the older age groups (39), making it harder for them to fulfill those needs and more likely to have unmet mobility needs. Compared to the older age groups, the “young–old” may also be less likely to have supports in place, such as through government funded home care services (40), increasing their likelihood of having unmet mobility needs. Surprisingly, sex is not associated with unmet mobility needs. Although international studies have reported inconsistent results in this regard (30, 31), the expectation that women are more likely to have unmet mobility needs is supported by their tendency to cease driving earlier than men (41), self-regulate their driving more than men (42), and experience more travel difficulties than men (32). However, previous research has also shown that women are more educated, independent, and self-sufficient now than previous generations (43), and, thus, they may find ways to fulfill their mobility needs regardless.

All health and wellbeing variables were independently associated with having unmet mobility needs. This is telling as, together, they capture various dimensions of health and wellbeing (35), both objectively—i.e., presence or absence of a long-term condition and physical limitation—and subjectively—i.e., appraisal of general health and psychological health using well-validated measures (44–47). As health issues can impede all means of mobility (modes of transportation) (48), the association between unmet mobility needs and having lower levels of self-rated health, having a long-term condition, being limited in everyday physical activities, and experiencing a higher level of distress is not surprising. In fact, a systematic review on the unmet mobility needs of older people noted that, despite ambiguity across the literature regarding other predictors, health issues are consistently reported to affect older people's mobility needs (29).

Regarding the built environment and transportation variables, being unlicensed, having decreased public transport ability, and residing in major cities were each independently associated with unmet mobility needs. The link between being unlicensed and having unmet mobility needs is consistent with previous studies that have examined this relationship specifically (9, 31), or the relationship between unmet mobility needs and other indicators of car access/use such as vehicle ownership (32) or driving frequency (30). The link between decreased public transport ability and unmet mobility needs is expected, as, similar to license ownership, public transport ability is indicative of having a particular transport mode at one's disposal, which increases one's ability to fulfill mobility needs. As health affects the ability to take public transport (49), one might expect the effect of public transport ability to disappear after adjusting for the health and wellbeing variables. However, this is not the case, suggesting that this “public transport ability” variable captures something other than health status. One explanation might be found in a study of older people's relationship to public transport, which explored how individuals differed in the extent to which they take ownership over their mobility resources (i.e., elements of their life that enable mobility such as health) and “convert” them into mobility capabilities (i.e., resources that can be deployed to support mobility) (50). It found mobility *capabilities*, not mobility *resources* alone, to lead to public transport use. In other words, it is not people's health *per se* that affects their ability to take public transport, but rather their evaluation of their health. Thus, this “public transport ability” variable may capture that evaluative mechanism (relating health specifically to public transport) while the health and wellbeing variables capture actual health (in general).

The finding that residence in major cities is associated with unmet mobility needs contrasts with findings from Finland (9) and the United States (30), showing an effect in the opposite direction, as well as that from Norway (31) and England (32), showing no location effect after adjustment. Our findings might be explained by the possibility that older people residing outside major cities have adjusted their out-of-home mobility needs to match the availability of amenities in their location. This leads them to have fewer out-of-home mobility needs compared to their urban dwelling counterparts, making it easier for them to fulfill those needs and resulting in them being less likely to have unmet mobility needs. A caveat to our finding, though, is that the residential location variable might be too coarse of a measure, having only three broad values and acting as a proxy for several pieces of information including urban connectivity, public transport availability, and availability of destinations. For example, residents of the City of Sydney, all of whom would be considered to reside in a “major city”, experience much variation in public transport service levels depending on the specific suburb in which they reside (51).

This study demonstrates that realized mobility, the focus of travel behavior research, and unmet mobility needs are two separate phenomena. In other words, a person or population may be “highly” mobile yet still have unmet mobility needs, as evidenced here. The finding that 96% of participants left home in the last two weeks may seem to suggest that out-of-home mobility is not a problem in this group. However, as other findings demonstrate, this is not the case. In some respects, the use of a 2 week time frame for calculating out-of-home mobility conceals the possibility that unmet mobility needs exist, as a high prevalence (96%) is more likely to be calculated within a wide time frame and the higher the prevalence of out-of-home mobility, the less attention is paid to unmet mobility needs. Two weeks could be considered too wide a time frame if one reflects on whether leaving home once in 2 weeks is typical of or sufficient for most people. Thus, this finding should not be overstated. This observation speaks to the limitation of using data from a routine survey, as questions are not fit-for-purpose.

This limitation also extends to the outcome measure, which, as it is operationalized, requires participants to hold two pieces of information in their heads (how often they would like to leave home and how often they actually leave home) and make a comparison between the two. The cognitive effort required to perform such a mental task may reduce the accuracy of the response (52). And this may explain why the prevalence of having unmet mobility needs among participants (12%) is lower than what is expected from the literature (~30%) (29). In contrast, a similar study in Birmingham, UK operationalized unmet mobility needs as “whether there are times when [you] cannot make the trips wanted” (48). It is conceivable that many would answer in the affirmative given the wording of the question. Thus, the operationalization of the outcome measure in this study may result in a conservative estimate of the extent of unmet mobility needs in the older Australian population. Regardless, there is a distinct pattern regarding the characteristics of older Australians most likely to report unmet mobility needs. A strength of this study is the inclusion of a range of measures covering different aspects of older people's mobility, as informed by the literature (35, 36), and the analysis of such measures on a large random sample of older Australians. However, as the survey was cross-sectional in nature, all factors were measured simultaneously, precluding inferences about the temporal link between predictor variables and the outcome of having unmet mobility needs.

Several conclusions arise from the findings. The first conclusion is that equity needs to be an explicit consideration, as older people with fewer personal resources are more likely to have unmet mobility needs. This is evidenced by our findings of higher odds of unmet mobility needs among older people with lower income, diminished health, and fewer transport resources¾ by virtue of being unlicensed or having decreased public transport ability. Notably, diminished health refers not only to physical ability (i.e., whether limited in everyday physical activities), but also to general health and wellbeing (i.e., self-rated health) and psychological health (i.e., level of distress). A second conclusion is that it is health more so than age that is predictive of unmet mobility needs. This is evidenced by the effect size of age being smaller than that of the health and wellbeing variables, supporting previous findings that the health of older people varies widely even among those of the same age group (53). The association between health and unmet mobility needs cannot be understated, as those with poor self-rated health have approximately four times (OR 4.30, 95% CI 3.06–6.04) the odds of having unmet mobility needs compared to those with excellent or very good self-rated health. The implication of this is that efforts to support older people's mobility cannot assume a one-size-fits-all approach and instead needs to be targeted to and inclusive of older people of all levels of health. Further, there is a need to improve the accessibility of cities and communities, such that public transport becomes a feasible option and residential location will no longer be a determining factor in having unmet mobility needs. This also helps to ensure that older people, regardless of their health status, are able to move about the city. This is especially important given Australia and many countries' commitment to aging in place, which prioritizes older people's continued residence in the community rather than in residential aged care.

Although studied internationally, unmet mobility needs have received limited attention in Australia due to a predominant focus on travel behavior and indicative transport disadvantage. By examining unmet mobility needs, this study directly addresses the issue of out-of-home mobility difficulty among older people by estimating its scale and identifying its predictors. Understanding the scale of the issue can inform policy interventions that aim to facilitate a minimum level of fulfilled mobility needs (3, 31). Understanding the predictors can inform policy interventions to be more effective, by considering the dimensions of the issue.

## Data availability statement

The data analyzed in this study is subject to the following licenses/restrictions: availability limited to subscribing organizations. Requests to access these datasets should be directed to Australian Bureau of Statistics.

## Author contributions

Conceptualization and methodology: TM, CK, and RI. Software, formal analysis, investigation, data curation, writing—original draft preparation, visualization, and project administration: TM. Writing—review and editing and supervision: CK and RI. All authors contributed to the article and approved the submitted version.
